# High‐throughput sequencing and fatty acid profile analyses of the Black Amur bream (*Megalobrama terminalis*) reveal variation in dietary niche associated with geographic segregation

**DOI:** 10.1002/ece3.11226

**Published:** 2024-04-16

**Authors:** Yaqiu Liu, Xinhui Li, Weitao Chen, Guangpeng Feng, Fangchan Chen, Jie Li, Qiong Zhou

**Affiliations:** ^1^ Key Laboratory of Freshwater Animal Breeding, Ministry of Agriculture and Rural Areas, College of Fisheries Huazhong Agricultural University Wuhan China; ^2^ Engineering Research Center of Green Development for Conventional Aquatic Biological Industry in the Yangtze River Economic Belt Ministry of Education Wuhan China; ^3^ Pearl River Fisheries Research Institute Chinese Academy of Fishery Sciences Guangzhou China; ^4^ Guangzhou Scientific Observing and Experimental Station of National Fisheries Resources and Environment Guangzhou China; ^5^ Jiangxi Institute for Fisheries Sciences, Poyang Lake Fisheries Research Centre of Jiangxi Province Nanchang China; ^6^ Guangzhou Qianjiang Water Ecology Technology Co. Ltd Gaungzhou China

**Keywords:** 18S rDNA, dietary niche, fatty acids, feeding strategy, geographic segregation, *Megalobrama terminalis*

## Abstract

Fish dietary niche is a core focus, and it reflects the diversity of resources, habitats, or environments occupied by a species. However, whether geographic segregation among different populations triggers dietary diversification and concomitant fish niche shift remains unknown. In the present study, we selected the Black Amur bream (*Megalobrama terminalis*) is a migratory fish species that plays an important role in the material transfer and energy cycling of river ecosystems, inhabiting southern China drainage with multiple geographic populations. Here, we utilized the combined analyses of 18S rDNA high‐throughput sequencing in fish gut contents and fatty acid (FA) in muscle tissues to evaluate potential spatial patterns of habitat and resource use for *M. terminalis* in three rivers of southern China. Our results showed that prey items of the Xijiang (XR) population (Pearl River) exhibited the highest species diversity and richness among the three geographic populations. Moreover, diet composition of *M. terminalis* was affected by spatial differences associated with geographic segregation. Analyses of FA biomarkers indicated that the highest levels of C16:0, C18:3n‐3, and C18:2n‐6c were found in Wanquan (WS) population (Wanquan River). The XR population exhibited a distinct FA profile characterized by higher amounts of arachidonic acid (ARA) and docosahexaenoic acid (DHA). The Moyang (MY) population (Moyang River) acted as the linkage between WS and XR populations and consisted of middle levels of saturated FAs (SFAs) and polyunsaturated FAs (PUFAs). The XR population displayed a greater FA niche width compared with WS population. Furthermore, we observed a close positive relationship between the niche width and α‐diversity indices of dietary resources for FA proflies. Our study provides valued information to develop different conservation strategies among different populations and improve fisheries management for *M. terminalis* and other endemic species in local rivers.

## Graphical abstract

This study provides valued viewpoints for understanding the variations in feeding strategy and dietary niche among geographically segregated populations of *Megalobrama terminalis*. Our results can be used to develop conservation strategies and improve the fisheries management of Black Amur bream or other endemic fish species.

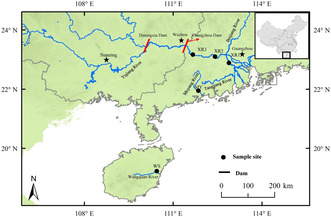

## 1 INTRODUCTION

Fish is the most diverse group of vertebrates on the earth, with over 34,000 species across a wide spectrum of habitats, physiology, and ecological strategies (Faircloth et al., 2013; Liu, Li, Li, & Li, 2022). Exploring the effects of environmental variables (e.g., geographic segregation and climate change) on the morphological characteristics and dietary niche of fish, and genetic structure of fish species diversity have received increasing attention globally. Of them, dietary niche is a core focus, and it reflects the diversity of resources, habitats, or environments occupied by a species (Pastore et al., 2021). Numerous studies show that characterizing the dietary requirements of organisms is essential to identify the trophic interactions, food webs, niche partitioning, and predator–prey relationships (Amarasekare, 2008; Dufy et al., 2007; Hoenig et al., 2020). Environmental factors play pivotal roles in influencing the prey abundance of fish in rivers and lakes (Bornette & Puijalon, 2011). For instance, river discharge and velocity are regarded as important way of controlling rivers in macrophyte colonization, establishment and persistence (Franklin et al., 2008). Moreover, geographic variations in the fish feeding strategy can provide important information on fish trophic diversification and adaption to natural habitat (Lesser et al., 2020). When habitat environment of fish tends to diversify, their prey items also produce distinct differentiation for the sake of environmental adaption. Therefore, quantifying the diet composition of fish in aquatic environment is important, as this can help reveal adaptative strategies of fish (Waraniak et al., 2019).

Predator–prey interactions are usually assessed by examining the diet items that remain in dissected gut and/or fecal samples through traditional morphology‐based methods (Corse et al., 2010; Evans et al., 2021; Pompanaon et al., 2012). However, quantifying decayed components in gastrointestinal contents proves to be difficult and can be only addressed when a diet component is identified prior to digestion (Buckland et al., 2017; Bunch et al., 2021). In recent studies, the method of DNA‐based species identification has been applied to improve the accuracy of diet identification in fish (Zhang et al., 2022). High‐throughput sequencing technique can be able to identify the species of gastrointestinal contents, and proves to overcome some shortcomings that occur in traditional morphology‐based methods (Watanabe et al., 2021). Based on the technique of high‐throughput DNA sequencing, researchers make a more effectively evaluation on prey assemblages, and then quantify trophic interactions (Casey et al., 2019; Hoenig et al., 2020). Specifically, high‐throughput DNA sequencing analysis is widely utilized to illuminate the diet composition of birds (Jedlicka et al., 2017) and bats (Vesterinen et al., 2013), whereas few studies focus on freshwater fish.

Fatty acids (FAs) are regarded as important component of aquatic organisms, generally accounting for 2%–15% of their dry weight (Wang, 2008). It has been reported that over 70 FAs are routinely detected within an aquatic organism (Budge et al., 2002; Iverson, 2009). For aquatic organisms, n‐3 and n‐6 series of polyunsaturated FAs can only be obtained from the diet and cannot be synthesized by themselves, namely essential FAs (Wang, 2008). These FAs are highly conservative during the trophic transfer along food chains, which allow them to distinguish the interspecific resource utilization models. Therefore, FA analyses are considered as a valid method for identifying foraging patterns and food‐web dynamics based on the assumption that lipids break down into their individual FAs and get incorporated with little alteration into the tissues of consumers (Chavarie et al., 2020; Iverson, 2009). Polyunsaturated FAs (e.g., eicosapentaenoic acid and docosahexaenoic acid) are usually utilized as important dietary biomarkers and applied to evaluate the dietary niche of various aquatic animals (Hu et al., 2022; Kabeya et al., 2018; Vasconi et al., 2015). Some signature FAs in muscle tissues can reflect the trophic levels of fish through feeding activities (Chavarie et al., 2020). Moreover, FAs can be used to determine the pattern of dietary niche differentiation as well as the degree of specialization and changes in feeding habits (Sardene et al., 2016).Thus, identifying the FA profiles of fish can help understand the trophic shift and trophic relationship of fish.

The Black Amur bream (*Megalobrama terminalis*) is a medium‐sized cyprinid fish and regarded as one of the most commercial fish species in southern China (Liu, Li, Li, Li, et al., 2021). The biomass of *M. terminalis* accounts for nearly 44.1% in all fish catches in the middle and lower reaches of the Pearl River basin (Liu et al., 2020). The drainage systems in Southern China are complex and diverse, and they have experienced numerous river alteration events in the past three decades. Geographical isolation caused by sea level fluctuations makes a profound impact on the genetic structure of *M. terminalis* populations (Chen et al., 2020). Previous studies reveal that three genetic populations (the Pearl River, Moyang River, and Wanquan River in Hainan Island) of *M. terminalis* are identified, and adaptive differentiation has been demonstrated across different habitats (Chen et al., 2020; Liu, Li, Li, Zhou, et al., 2022). *Megalobrama terminalis* is an omnivorous fish species with strong digestibility and dietary plasticity (Liu et al., 2020). Therefore, *M. terminalis* is a good model for evaluating dietary niche differentiation and diversity among geographic populations. Generally, there is a close relation between fish diet and habitat environment (Borzone Mas et al., 2022; Meekan et al., 2022). We hypothesized that geographical isolation triggers trophic differentiation among different geographical populations of *M. terminalis*. To test this hypothesis, we attempted to (1) employ 18S rDNA metabarcoding to isolate and amplify DNA sequences from prey items in the fish gut of three geographical populations and (2) identify potential variations in feeding strategies and dietary niches of three populations via illuminating their FA profiles. This study provides valued viewpoints for understanding the variations in feeding strategy and dietary niche among geographically segregated populations of *M. terminalis*. Moreover, our results can be used to develop conservation strategies and improve the fisheries management of the Black Amur bream or other endemic fish species.

## 2 MATERIALS AND METHODS

### 2.1 Study area description and sample collection

The Pearl River, located in subtropical southern China, is the second largest river in China in terms of water discharge and has a total length, catchment area, and annual discharge of 2320 km, 450,000 km^2^, and 10,000 m^3^ s^−1^, respectively (Wang et al., 2014). The Moyang River is an inland river that originates from the Cloud Mountain and ultimately flows toward the South China Sea, with a total length, catchment area, and annual discharge of 199 km, 6091 km^2^, and 260 m^3^ s^−1^, respectively. The Wanquan River, located in the central and eastern part of Hainan Province (China), is the third longest river in the Hainan Island and has a total length, catchment area, and annual discharge of 163 km, 3683 km^2^, and 185 m^3^ s^−1^, respectively.

**FIGURE 1:**
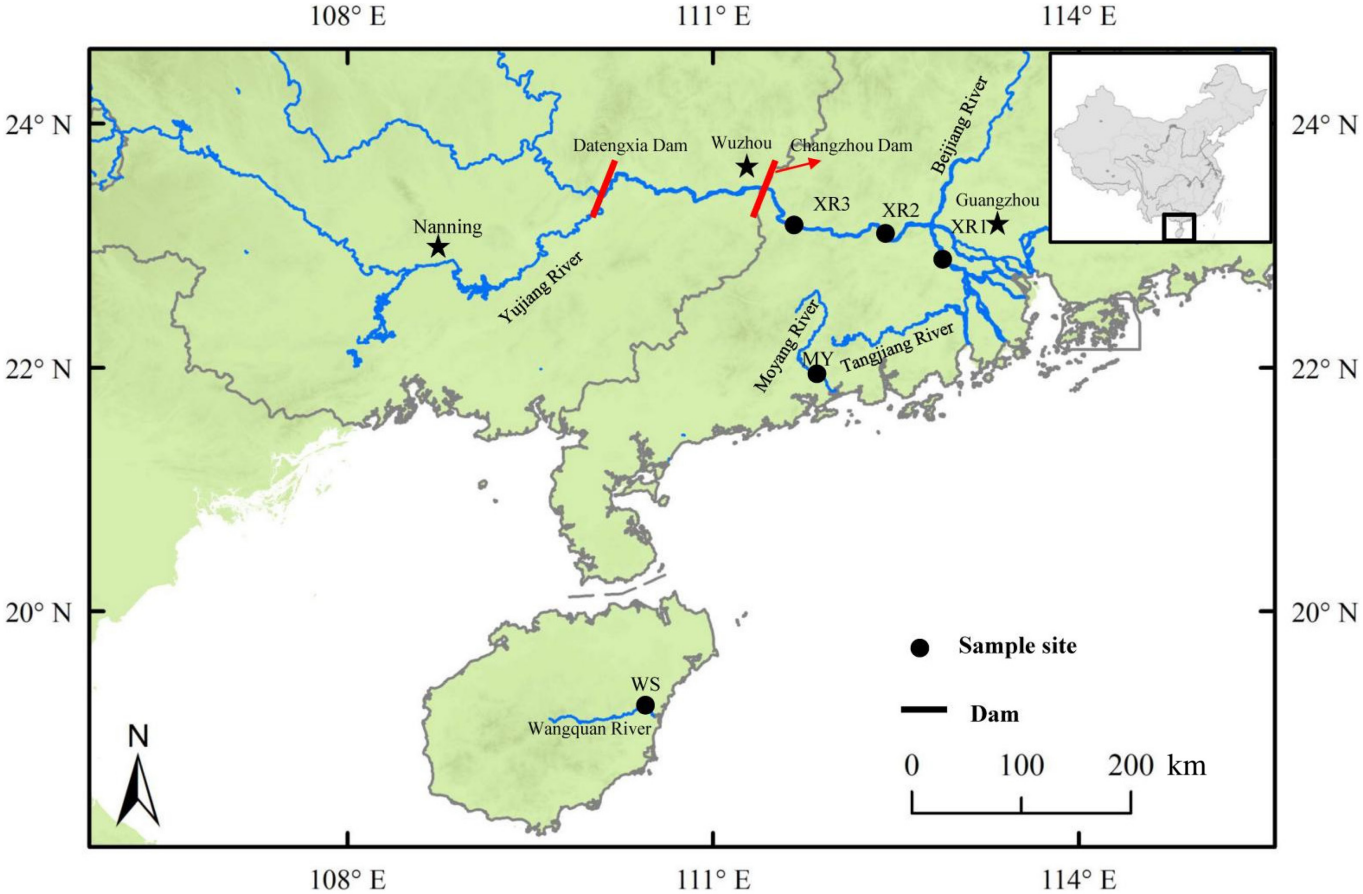
Sketch map showing the locations of sampling sites for *Megalobrama terminalis* and three geographic populations.

We collected fish samples from the Pearl River (XR), Moyang River (MY), and Wanquan River (WS) in July 2019 and January 2020. Locations of sampling sites and the characteristics of biology and environment are available in Figure 1 and Table 1. Fish sampling was carried out by using circular cast nets (15 m diameter, mesh size 4 cm). One hundred and twenty samples were collected (forty individuals of *M. terminalis* from each river). The standard body length (SL, to the nearest 1 mm), body weight (Wt, to the nearest 1 g), and fatness (*K* = 100 × W/L^3^) were measured for each individual (Table 1). All fish individuals were anesthetized with an overdose of MS 222 (3‐aminobenzoic acid ethyl ester methane sulfonate, Sigma, Germany), stunned, and then decapitated quickly. The surface of both fish body and instruments was wiped with 75% ethanol to avoid contamination, and, meanwhile, the instruments were sterilized before the dissection. Gut contents and dorsal white muscles of all fish individuals were swiftly dissected in liquid nitrogen and immediately transferred to an ultralow temperature freezer and preserved at −80°C until further analysis.

**TABLE 1:** Selected environmental and biological characteristics for the three geographical populations of *Megalobrama terminalis.*

Parameter	Population		
	WS	MY	XR
Temperature (°C)	24.2 ~ 30.1	19.5 ~ 29.1	18.9 ~ 28.7
Salinity (‰)	0.03 ~ 0.09	0.01 ~ 0.04	0.008 ~ 0.01
pH	7.7 ~ 8.1	7.9 ~ 8.2	7.8 ~ 8.2
DO (mg/L)	6.4 ~ 6.8	6.3 ~ 6.7	6.6 ~ 7.0
SL (mm)	214.5 ~ 249.5	223.6 ~ 268.5	241.5 ~ 275.6
Wt (g)	275.2 ~ 311.5	285.3 ~ 320.7	368.9 ~ 420.6
*K*	1.8 ~ 2.1	1.9 ~ 2.3	2.0 ~ 2.5

Abbreviations: MY, population in the Moyang River; WS, population in the Wanquan River; XR, population in the Pearl River.

### 2.2 DNA extraction and amplification

Total microbial genomic DNA of 12 fish samples random selected in each group was extracted through the cetyltrimethylammonium bromide (CTAB) method (Allen et al., 2006). The extracted DNA was assessed for the quality, integrity, concentration, and purity using 1% agarose gels, and then stored at −80°C until further test. For the analysis, the V9 hypervariable region of 18S rDNA genes was amplified by using specific primers (1380F − 1510R) with barcodes (Amaral‐Zettler et al., 2009). Total DNA in the gut contents of fish from different geographical populations was sent to Novogene Bioinformatics Technology Co., Ltd. (Beijing, China) for further sequencing analysis.

### 2.3 High‐throughput sequencing analysis

An Illumina NovaSeqPE250 (Illumina, USA) was utilized to generate sequencing libraries according to the manufacturer's recommended procedures. Paired‐end reads were allocated to respective samples by utilizing distinctive barcode. After that, the reads were truncated by removing the barcode and primer sequence. The truncated paired‐end reads were merged together using the FLASH software (version 1.2.7; Magocˇ & Salzberg, 2011).To obtain high‐quality clean tags, raw tags underwent quality filtering using specific filtering conditions. The operation referred to the QIIME (version 1.9.1) quality control process (Caporaso et al., 2010). The tags were compared against the Silva database (http://www.arb‐silva.de/) using the UCHIME algorithm to identify chimeric sequences (Edgar et al., 2011; Quast et al., 2013). The chimeric sequences were removed to obtain effective tags according to the method of Haas et al. (2011). Sequencing analysis was conducted for the operational taxonomic unit (OTU) clustering using Uparse (version 7.0.1; Edgar, 2013). Sequences with a similarity of 97% were categorized into the same OTU. Each OTU was then assigned a representative sequence and annotated with taxonomic information based on the Silva database and the Mothur algorithm (Schloss et al., 2009). To eliminate any potential bias caused by DNA contamination from the Black Amur bream, we excluded OTUs annotated with vertebrates (phylum Chordata) from the data analysis. This decision was based on previous research conclusion that the Black Amur bream predominantly feed on algae, zooplankton, and invertebrates (Lu, 1990). The relative abundance of each prey taxon at each sample was calculated by utilizing the sequence counts of each OTU and the annotated taxonomic information according to the methodology revealed by Deagle et al. (2019). The alpha diversity of OTUs for different geographical populations was calculated in four metrics (Shannon, Simpson, Chao1, and Ace indices) using Mothur v1.30.1 (Schloss et al., 2009).

### 2.4 Fatty acid analysis

Fatty acid methyl esters (FAMEs) of each sample from three populations (sample size: WS = 15, MY = 15, and XR = 30) were measured through the GAQSIQ method with minor modifications (GAQSIQ, 2008). A mixture of methanol and chloroform (2:1, v/v) was utilized to extract lipids from each tissue sample, and the lipid content was determined gravimetrically (Parrish, 1999; Rossi et al., 2006). The lipids were then trans‐esterified with boron trifluoride‐methanol and analyzed as FAMEs using a gas chromatograph/mass selective detector (7890B/5977A, Agilent Technologies, Santa Clara, CA, USA) equipped with an HP‐88 capillary column (60 m × 0.25 mm × 0.2 μm, Agilent Technologies; Hu et al., 2022). Methyl nonadecanoate (19:0) was used as an internal standard. Samples were injected in splitless mode using helium as carrier gas, with a thermal gradient from 125°C to 250°C, and a set auxiliary heater temperature of 280°C. We identified FAs by comparing their relative retention times with known standards and FA data in this study (GAQSIQ, 2008). The data were expressed as percentages of the total FAs present in samples.

### 2.5 Statistical analysis

We utilized a Scale‐Venn diagram to illustrate the shared and unique OTUs among different geographical populations. Furthermore, a principal coordinate analysis (PCoA) of the binary Jaccard distance was used to performe discrepancy between the diet compositions of different *M. terminalis* populations using Vegan packages. In addition, we utilized paired analysis of similarities (ANOSIM) to evaluate potential differences in the gut contents of three populations. R (version 4.0.2.) was used to conduct principal component analysis (PCA) on FA structure. We calculated the contribution rate and cumulative contribution rate of each principal component and plotted a scatter point diagram of the principal components. Trophic niches of the three populations were characterized by the standard elliptic area (SEA), based on the results of principal components analysis (PC1 and PC2). The R language SIAR software package was employed for the calculation of corrected standard ellipse area (SEAc) according to the methodology of Pedro et al. (2020). To evaluate the differences in alpha diversity, polyunsaturated fatty acid (PUFA), saturated fatty acid (SFA), monounsaturated fatty acid (MUFA), total fatty acid (TFA), EPA/DHA, and PUFA/SFA among the three populations, one‐way analysis of variance (ANOVA) was conducted using SPSS 28.0 (IBM, Chicago, USA). A *p*‐value below .05 was set as statistical significance. The relationships between the SEAc values and α‐diversity indices were investigated separately using linear regression analysis. A *p*‐value less than .05 was considered to indicate a significant pairwise relationship.

## 3 RESULTS

### 3.1 Diet composition

**FIGURE 2:**
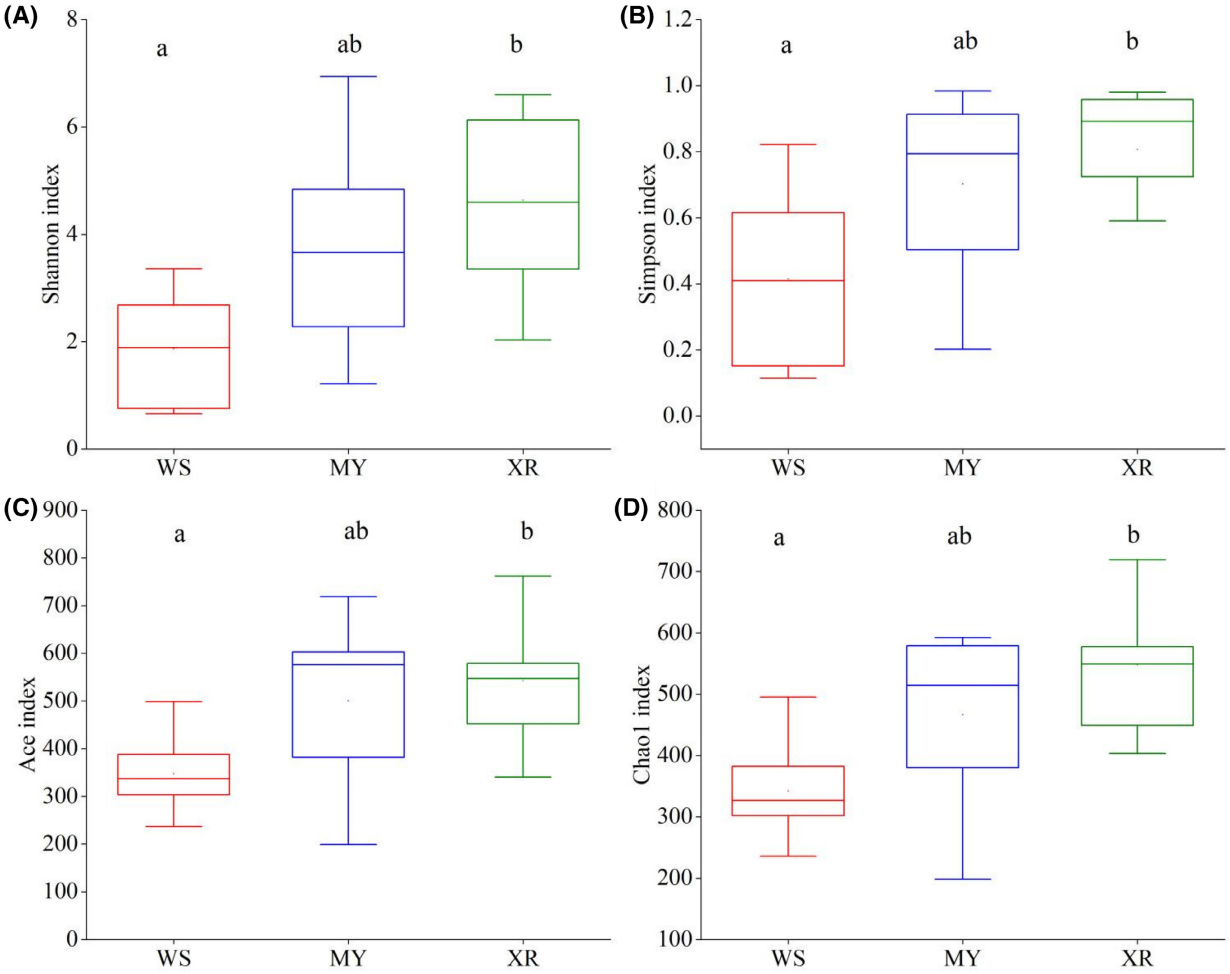
α‐Diversity of the diet composition from three populations of *Megalobrama terminalis*. (A)‐Shannon index; (B)‐Simpson index; (C)‐Ace index; (D)‐Chao1 index. Different lowercase letters indicate significant differences (*p* < .05). MY, population in Moyang River; WS, population in Wanquan River; XR, population in the Pearl River.

**FIGURE 3:**
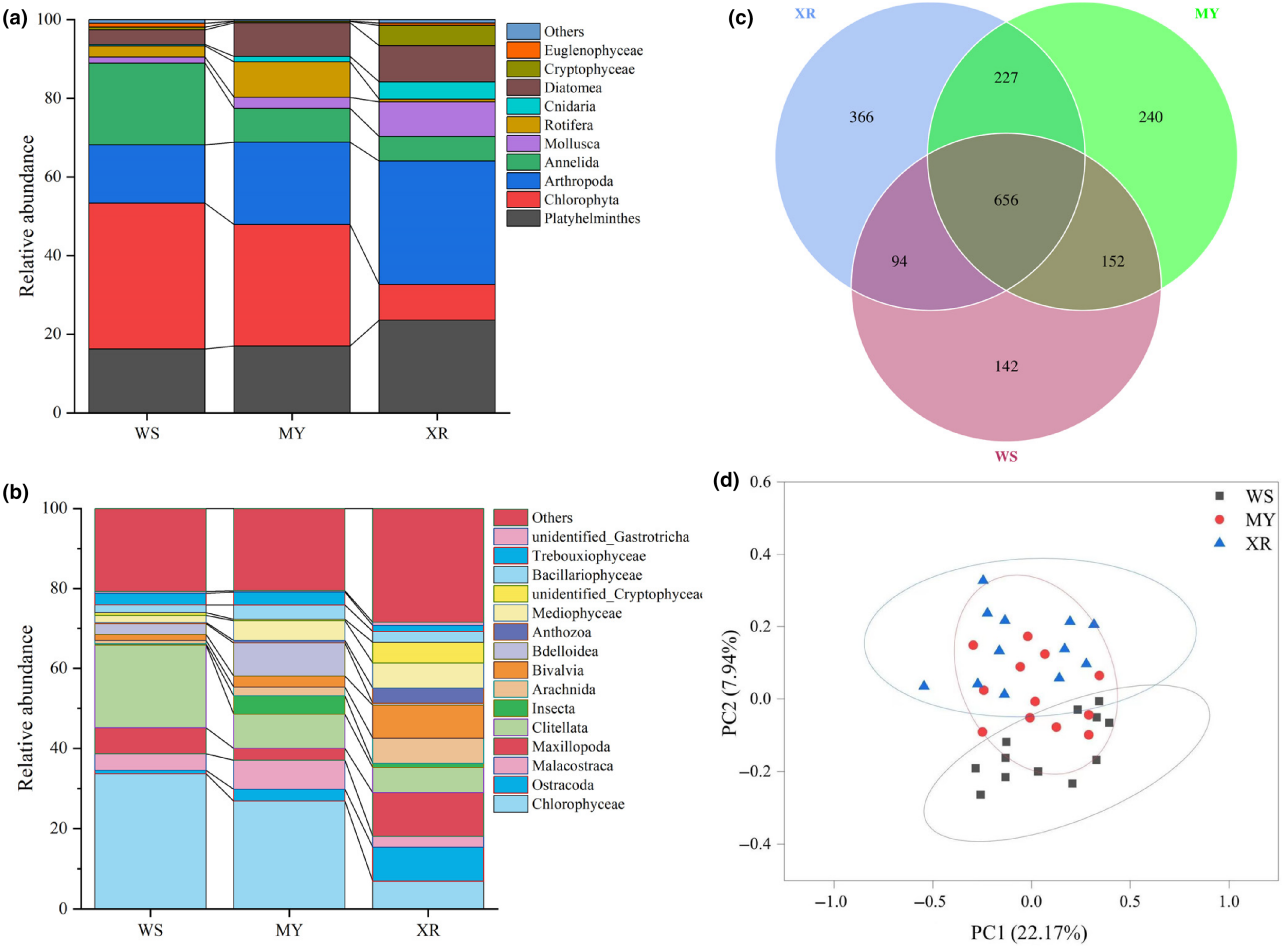
Mean relative abundances of taxa up to the phylum (a) and at the class (b) levels for the gut contents of *Megalobrama terminalis*; (c) Venn diagram displays the number of shared and unique OTUs between three geographical populations. (d) PCoA (binary Jaccard distance) plot for the diet composition of *M. terminalis* among the three populations. Samples of individuals are color‐coordinated according to different groups. MY, population in Moyang River; WS, population in Wanquan River; XR, population in the Pearl River.

We obtained a total of 98,047 quality‐filtered sequences from each sample. A total of 1877 OTUs were identified by grouping the sequences using a 97% sequence similarity cutoff. The WS population showed significantly lower alpha diversity and richness compared with the other two populations, and the XR population exhibited the highest alpha diversity and richness among the three geographic populations (*p* < .05, Figure 2). Our results showed that Chlorophyta (37.11%), Annelida (20.74%), and Platyhelminthes (16.27%) were the most abundant phyla in the diet of WS population, whereas those of the MY population were Chlorophyta (30.89%), Arthropoda (20.95%), and Platyhelminthes (17.05%; Figure 3a). Dominant phyla in the diet of XR population were Arthropoda (31.39%), Platyhelminthes (23.61%), and Diatoma (9.18%; Figure 3a). At a class level, Chlorophyceae (33.63%) and Clitellata (20.74%) in WS population showed higher relative abundances than those in the other two populations, whereas the classes Maxillopoda (10.87%), Ostracoda (8.44%), and Bivalvia (8.23%) were significantly abundant in XR population (Figure 3b). In samples from MY population, the Chlorophyceae (26.88%), Clitellata (8.52%), and Bdelloidea (8.29%) were significantly abundant classes (Figure 3b). Three geographic populations shared 656 OTUs, and the WS population exhibited fewer unique OTUs than the other two populations (Figure 3c). The number of common OTUs present in three populations was 656, and the number of unique OTUs for each group varied from 142 to 366 (Figure 3c). A separation of the community composition between WS and XR populations was shown, and the samples of MY population stood between those WS and XR populations (ANOSIM: *R* = .212, *p* = .014, Figure 3d). PCoA1 and PCoA2 explained 22.17% and 7.94% of the total variance, respectively, indicating that the community composition of fish diet was influenced by complex factors.

### 3.2 Fatty acid analysis

**FIGURE 4:**
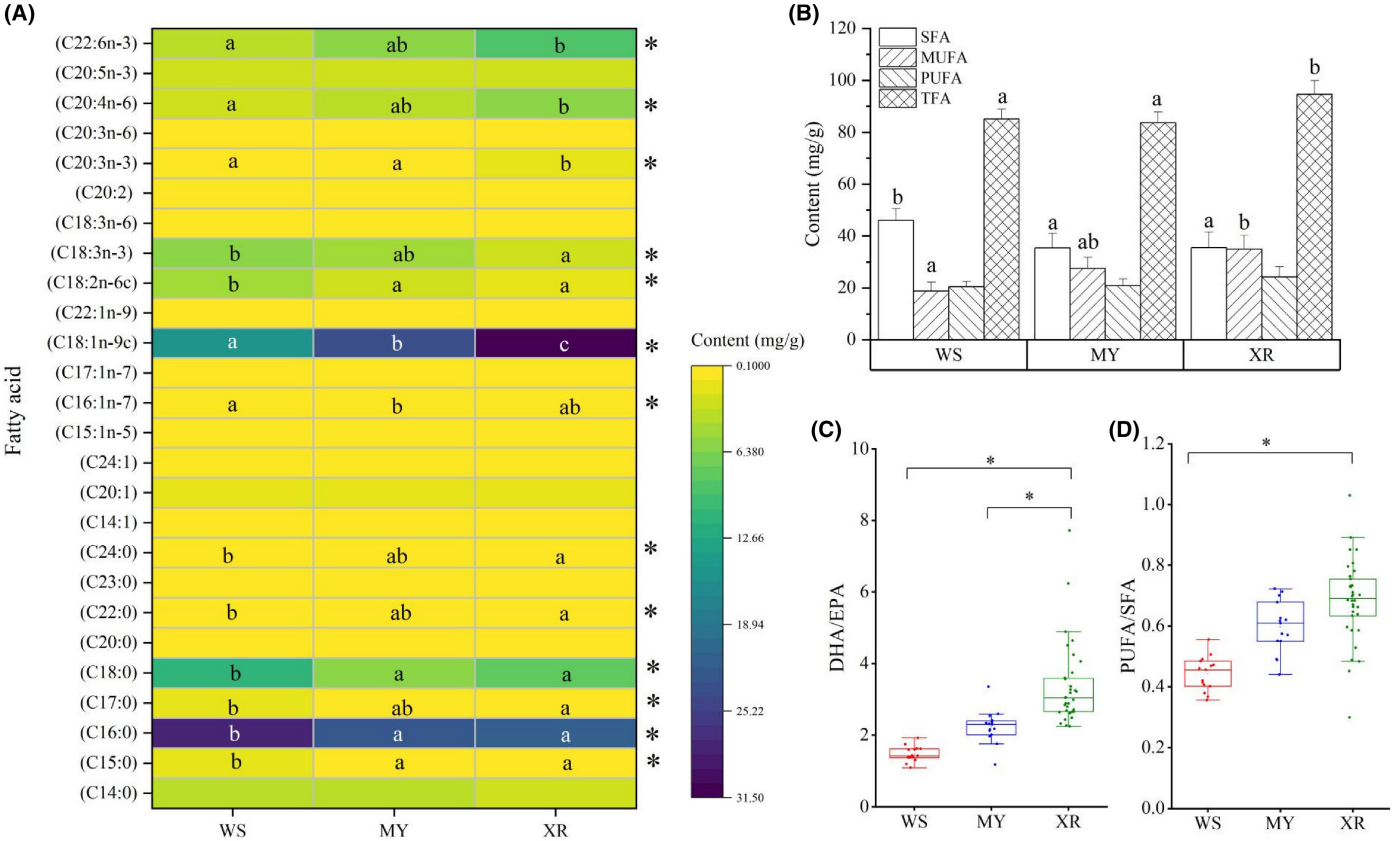
Fatty acid profiles analysis for three populations of *Megalobrama terminalis*. (A) The Heatmap presenting the abundance of fatty acid profiles in muscles of *M. terminalis* among three populations. Samples marked different capital letters indicate significant differences (a < b < c, *p* < .05) among different populations. (B) Bar plot indicating the abundance of SFA, TFA, MUFA, and PUFA for different populations of *M. terminalis*. (C) Box plot showing significant differences of DHA/EPA among three populations. (D) Box plot showing significant differences of PUFA/SFA among three populations. * indicate significant differences between two groups, *p* < .05. DHA (C22:6*n*‐3), docosahexaenoic acid; EPA (C20:5n‐3), eicosapentaenoic acid; MUFAs (C14:1, C15:1n‐5, C16:1n‐7, C17:1n‐7, C18:1n‐9c, C20:1, C22:1n‐9, C24:1), monounsaturated fatty acids; MY, population in Moyang River; PUFAs (C18:2n‐6c, C18:3n‐3, C18:3n‐6, C20:2, C20:3n‐3, C20:3n‐6, C20:4n‐6, C20:5n‐3, C22:6n‐3), polyunsaturated fatty acids; SFAs (C14:0, C15:0, C16:0, C17:0, C18:0, C20:0, C22:0, C23:0, C24:0), saturated fatty acids; TFA, total fatty acids; WS, population in Wanquan River; XR, population in the Pearl River.

A total of 26 FAs were identified for the three populations of *M. terminalis*. It consisted of nine saturated FAs (SFAs), eight monounsaturated FAs (MUFAs), and nine polyunsaturated FAs (PUFAs; Figure 4A). Significant variations in the proportions of main FA profiles were observed among three geographic populations. The abundance of six SFAs in WS population was higher than those in XR population (*p* < .05, Figure 4A). Total amount of SFAs in WS population was higher than that in MY and XR populations (*p* < .05, Figure 4B). Total amount of MUFAs in WS population was much lower than that in XR population (*p* < .05, Figure 4B). The XR and WS populations showed the highest and lowest abundance of (C18:1n‐9c), respectively, whereas the MY population had the highest abundance of (C16:1n‐7). Total amount of TFAs was the highest in XR population. The mean abundance of docosahexaenoic acid (DHA) in XR population was significantly higher than that in WS and MY populations (*p* < .05, Figure 4A). Moreover, the DHA/EPA ratio (1.46) in WS population was lower than those of MY (2.24) and XR (3.38) populations (Figure 4C), and the PUFA/SFA ratio (0.68) in XR population was higher than in WS population (0.44; Figure 4D).

### 3.3 Principal component analysis of fatty acid composition

**FIGURE 5:**
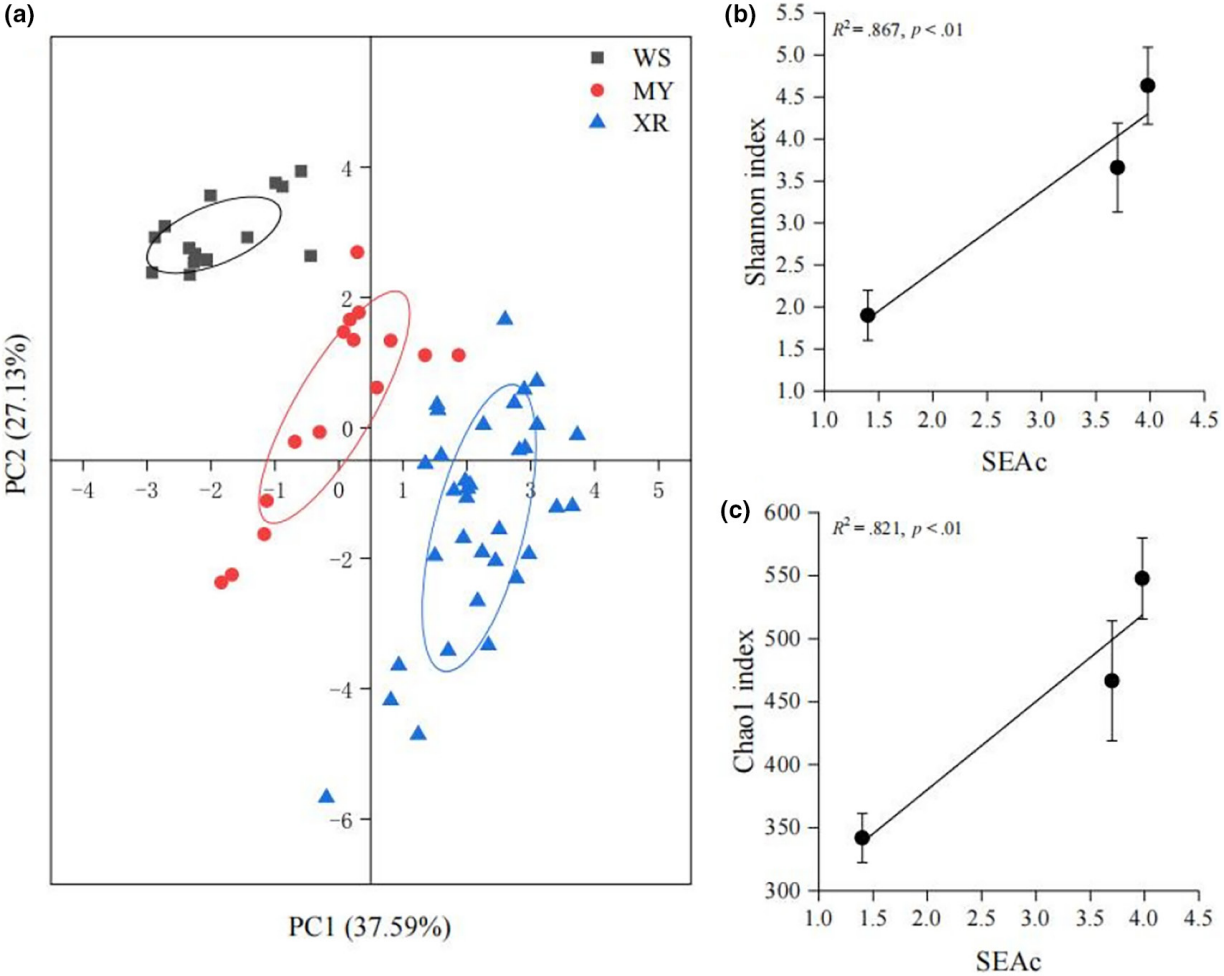
Dietary niche diversification analyses for three populations of *Megalobrama terminalis*. (a) Principal component analysis of fatty acid profiles among three populations. Linear regression analysis of the corrected ellipse area (SEAc) values and α‐diversity indeices ((b). Shannon index; (c), Chao1 index) of fish among three populations. MY, population in Moyang River; WS, population in Wanquan River; XR, population in the Pearl River.

According to the SIMPER analysis, specific FAs (C16:0, C18:0, C18:1n‐9c, ARA, and DHA) contributed 71.9% of the difference between the samples of WS and XR populations (Table 2). Meanwhile, the difference between the samples of WS and MY populations was primarily due to C18:1n‐9c, C16:0, C16:1n‐7, C18:0, and DHA, which contributed 65.4% of the dissimilarity (Table 2). It was found that C18:1n‐9c, C20:3n‐3, ARA, and DHA contributed to the dissimilarity between MY and XR populations. The XR population exhibited the greatest dietary niche width among three populations, whereas the WS population showed the lowest (Figure 5a). Significant positive correlations were detected between corrected ellipse area (SEAc) and the Shannon and Chao1 indices of diet compositions (Figure 5b,c).

**TABLE 2:** SIMPER analysis of fatty acid profiles among three populations of *Megalobrama terminalis.*

Item	MY versus XR contribution	WS versus XR contribution	MY versus WS contribution
C14:0	0.011	0.015	0.019
C15:0	0.005	0.015	0.011
C16:0	0.127	0.198	0.175
C17:0	0.008	0.054	0.039
C18:0	0.018	0.078	0.065
C20:0	0.007	0.007	0.007
C22:0	0.045	0.007	0.017
C23:0	0.004	0.006	0.011
C24:0	0.063	0.009	0.018
C14:1	0.018	0.005	0.011
C20:1	0.006	0.003	0.008
C24:1n‐9	0.003	0.006	0.008
C15:1n‐5	0.014	0.005	0.011
C16:1n‐7	0.010	0.018	0.075
C17:1n‐7	0.008	0.010	0.008
C18:1n‐9c	0.184	0.217	0.261
C22:1n‐9	0.004	0.005	0.010
C18:2n‐6c	0.097	0.016	0.015
C18:3n‐3	0.026	0.006	0.014
C18:3n‐6	0.027	0.004	0.011
C20:2	0.015	0.002	0.006
C20:3n‐3	0.092	0.047	0.041
C20:3n‐6	0.039	0.004	0.010
C20:4n‐6	0.064	0.105	0.050
C20:5n‐3	0.024	0.035	0.022
C22:6n‐3	0.082	0.121	0.078

Abbreviations: MY, population in the Moyang River; WS, population in the Wanquan River; XR, population in the Pearl River.

## 4 DISCUSSION

### 4.1 Diet composition and diversity

Knowledge of the association between geographic population distribution and diet composition is fundamental for understanding the environmental adaption and ecological evolution of Black Amur bream. Greene et al. (2020) show that there is a close relationship between dietary niche and gut microbiome of hosts. It has been reported that geographic isolation forms relatively independent gut microbiome for different populations of *M. terminalis* (Liu, Li, Li, Zhou, et al., 2022). Until now, however, very few studies reported the difference of dietary diversity among different populations of *M. terminalis*.

Our study investigated the dietary niche and diversity of *M. terminalis* caused by geographic segregation using 18S rDNA metabarcoding and multiple FA analysis. We found significant differences in diet composition and FA profiles among three geographic populations, possibly driven by habitat, site‐specific dietary sources and biogeochemical processes (Hoenig et al., 2020; Xie et al., 2020). Based on the high‐throughput sequencing analysis of fish gut contents, prey items in XR population displayed the highest diversity and richness among the three populations (Figure 2), indicating that food resources utilized by the XR population had higher diversification. The relative abundance of plant resources in fish gut contents of WS population was higher than those in MY and XR populations. Moreover, the relative abundance of animal resources in fish guts of XR population was much higher than that of WS population. Habitat differentiation of fish populations is regarded as one of the most important factors for the discrepancy of dominant prey items in fish (Bade et al., 2014; Sun et al., 2021). Zooplankton and zoobenthos are primary food items for fish in rivers, and their abundances significantly correlate with physicochemical factors such as water temperature, dissolved oxygen, transparency, phosphate, and copper. (Wang et al., 2016). Furthermore, the results of PCoA analysis indicated that geographical location was closely associated with fish diet composition (Figure 3d). Previous researches show that *M. terminalis* is an omnivorous fish with relatively high digestive plasticity (Liu et al., 2020; Liu, Li, Li, & Chen, 2021). It seems that *M. terminalis* can utilize different food sources at different habitats when available. High diet diversification in XR population was closely related to wide range of habitat and abundant food sources. Recent studies have shown that geographic variation in the feeding strategy of animals can provide valuable information on animals' trophic diversification (Gong et al., 2020). Moreover, genotypes (e.g., some olfactory related genes) alteration was considered as another factor for the shift of fish diet composition (Liu, Chen, et al., 2021). Thus, long‐term geographic isolation leads to variations in environmental factors and genotypes, thereby triggers remarkable differentiation of diet composition for *M. terminalis*. Chilton and Muoneke (1992) suggested that food availability and biological environment (e.g., other fish in the community) are important affected factors for fish dietary differentiation. In addition, changes in habitat type and food sources may relate to the genetic structure of different *M. terminalis* populations. A significant correlation between fish genetic distance and dietary differentiation has already been reported (Pilot et al., 2012).

### 4.2 Fatty acid profile differentiation

FA profile analysis revealed a clear intraspecific differentiation in habitat use (Figure 5a), and this was consistent with the results of 18S rDNA sequencing analysis for diet composition. Research on sardine illuminates that significant geographical differences of FA composition are associated with the difference in the diet of sardine (Garrido et al., 2008). The highest levels of C16:0, C18:3n‐3, and C18:2n‐6c in WS populations reflected the characteristics that freshwater food webs were supported primarily by Chlorophyceae (Vasconi et al., 2015; Zhu & Cao, 2002). By contrast, the XR population had distinct FA profiles that included high amounts of ARA and DHA, which are characteristic of zoobenthos and zooplankton, respectively (Kelly et al., 2009; Stowasser et al., 2009). Higher level of DHA in XR populations implied high contribution of carnivorous animals to *M. terminalis* (Li et al., 2022; Meyer et al., 2019). In addition, our result showed that DHA/EPA ratio in WS population (1.46) was much lower than that in MY (2.24) and XR (3.38) populations. External nutrients of fish selectivity are pivotal for the differences of fish FAs (Gomes et al., 2016; Pond, 2009). Recent studies showed that the DHA/EPA ratio is proportional to the trophic level of fish (Dalsgaard et al., 2003; Méndez‐Da Silveira et al., 2020). Due to the influence of geographical isolation, trophic levels of different *M. terminalis* populations significantly differentiated. Specially, we found that abundance of SFAs and PUFAs in MY population fell in between WS and XR populations, in accordance with the genetic and geographic differentiation among three populations (Chen et al., 2020). Furthermore, XR population exhibited a higher PUFA/SFA ratio than WS and MY populations, suggesting that XR population possessed a wider range of feeding ground, and had more abundant PUFA. Recent studies considered that trophic transfer of PUFA in food webs depends upon taxon‐specific feeding strategies and selective metabolism of FA in consumer (Strandberg et al., 2015). Our another study indicated that *M. terminalis* is an omnivorous fish in the middle and lower water layers, with flexible feeding strategy and strong adaptability for diverse habitat environments (Liu et al., 2020). The difference of habitat environment associated with geographical isolation may significantly affect the feeding strategy of *M. terminalis* for different populations. Based on site‐specific FA biomarkers, spatial patterns of dietary sources revealed diverse feeding strategies in *M. terminalis*, which can help enhance feeding success and facilitate the adaptation to varying environmental conditions.

### 4.3 Dietary niche diversification

The XR population displayed a larger FA niche width among three populations (Figure 5a), implying higher utilization of food and habitat (Gong et al., 2020). However, the FA niche width of WS population was more narrow than those of the other two populations in that the habitat area of WS population was smaller and the demand of food resources was more simple. Observed feeding patterns accord with the results of PCA for FA profiles, possibly indicating more generalized feeding strategies that rely on freshwater food webs (Figure 5a). The differentiation of trophic niches can be a sign of interspecific feeding differences and habitat differentiation (Gao et al., 2018). Interspecific spatial isolation is an effective method of alleviating competition for food resources (Lin et al., 2020). Interestingly, we observed that the FA niche width and α‐diversity indices of dietary composition for *M. terminalis* showed a close positive relationship (Figure 5b,c), suggesting that the diversification in fish diet significant affected the tropic niche width of *M. terminalis*. It has been found that trophic niche widths changes of different *M. terminalis* populations are important adaptive way to their stable existence in food webs and even aquatic ecosystems (Layman et al., 2007). The distribution pattern of food resources among different species of *M. terminalis* is dynamic and closely related to the size and abundance of food biological resources in their habitat waters (Olson et al., 2015). In this study, XR population had a higher diversity of feeding sources than WS and MY populations, indicating that the degree of inter‐individual feeding overlap was lower, resource allocation was more refined, and food utilization was higher than the other populations. Correlational study has shown that there is a high overlap of feeding time and nutrient niche among coexisting individuals, which reduces the resource utilization efficiency of populations (Carrasson & Cartes, 2002).

## 5 CONCLUSION

Our study illuminated significant differences in diet composition and FA profiles of *M. terminalis* among the three geographic populations (Pearl River‐XR, Moyang River‐MY, Wanquan River‐WS). Prey items in XR population had the highest diversity and richness among three populations. Moreover, the diet composition of *M. terminalis* was affected by spatial differences associated with geographic segregation. The XR population showed a larger FA niche width than WS population. The FA niche width and α‐diversity indices of dietary resources exhibited a close positive relationship. Habitat environment and the diversity of prey items triggered variation in feeding strategies among three geographic populations. Our study provides valued viewpoints for developing conservation strategies of different populations and improving fisheries management of *M. terminalis* and other endemic species in the rivers. Nevertheless, it is necessary to clarify how geographic variation effects fish feeding behavior and physiology. Therefore, exploring the geographic differentiation triggers the trophic transfer of FA in fish populations should be considered in a future study.

## AUTHOR CONTRIBUTIONS

**Yaqiu Liu:** Data curation (lead); funding acquisition (equal); software (lead); validation (lead); writing – original draft (lead). **Xinhui Li:** Funding acquisition (equal); supervision (equal). **Weitao Chen:** Investigation (equal). **Guangpeng Feng:** Investigation (equal). **Fangchan Chen:** Investigation (equal). **Jie LI:** Funding acquisition (equal). **Qiong Zhou:** Formal analysis (equal); supervision (lead); writing – review and editing (lead).

## CONFLICT OF INTEREST STATEMENT

Tha authors have declared no conflict of interest.

## Data Availability

DNA sequences: NCBI SRA BioProject ID PRJNA979942.
